# External validation of scores proposed for estimation of survival probability of patients with severe adult respiratory distress syndrome undergoing extracorporeal membrane oxygenation therapy: a retrospective study

**DOI:** 10.1186/s13054-015-0875-z

**Published:** 2015-12-01

**Authors:** Stephanie Klinzing, Urs Wenger, Peter Steiger, Christoph Thomas Starck, Markus Wilhelm, Reto A Schuepbach, Marco Maggiorini

**Affiliations:** 1 Surgical Intensive Care Medicine, University Hospital of Zurich, Raemistrasse 100, CH-8091 Zurich, Switzerland; 2 Medical Intensive Care Unit, University Hospital of Zurich, Raemistrasse 100, CH-8091 Zurich, Switzerland; 3 Department of Cardiac and Vascular Surgery, University Hospital of Zurich, Raemistrasse 100, CH-8091 Zurich, Switzerland

**Keywords:** Adult Respiratory Distress Syndrome, Preserve Score, Peak Inspiratory Pressure, ECMO Support, Receiver Operating Curve Analysis

## Abstract

**Introduction:**

This study was designed as an external validation of the recently proposed Predicting Death for Severe ARDS on V-V ECMO (PRESERVE) score, The respiratory extracorporeal membrane oxygenation survival prediction (RESP) score and a scoring system developed for externally retrieved patients on extracorporeal membrane oxygenation (ECMO) at our institution. All scores are proposed for the estimation of survival probability after ECMO treatment for severe adult respiratory distress syndrome.

**Methods:**

Data from 51 patients (2008 to 2013) were analyzed in this retrospective single-center study. A calculation of an adapted PRESERVE score, the RESP score as well as the score developed for externally retrieved ECMO patients was performed.

**Results:**

Seventy one percent of patients received veno-venous (v-v) and 29% venous-arterial (v-a) ECMO support during the study period. Overall survival at 6 months was 55%, with a 61% survival rate for v-v cannulated patients and a 40% survival rate for v-a cannulated patients. The PRESERVE score discriminated survivors and non-survivors with an area under the curve of 0.67 (95% CI 0.52 to 0.82, *P* = 0.03). Analyzing survival prediction according to cannulation modus, the PRESERVE score and the RESP score significantly predicted survival for patients on v-v ECMO with an area under the curve of 0.75 (95% CI 0.57 to 0.92, *P* = 0.01) and 0.81 (95% CI 0.67 to 0.95, *P* = 0.035), respectively, while the scoring system developed for externally retrieved ECMO patients failed to predict survival in our study population. All scores failed to predict mortality for patients on v-a ECMO.

**Conclusion:**

Our single-center validation confirms that the proposed PRESERVE and RESP score predict survival for patients treated with v-v ECMO for severe adult respiratory distress syndrome.

**Electronic supplementary material:**

The online version of this article (doi:10.1186/s13054-015-0875-z) contains supplementary material, which is available to authorized users.

## Introduction

Adult respiratory distress syndrome (ARDS) still has mortality rates between 45 and 55% [1]. Therapy remains supportive, with a proven survival benefit for lung protective ventilation [2] and prone positioning [3]. Despite advanced supportive procedures, including high-frequency ventilation, nitric oxide (NO) inhalation and steroids, a subgroup of patients remains severely hypoxemic [4].

Extracorporeal membrane oxygenation (ECMO) support as a rescue therapy has gained renewed interest since the encouraging, though debated, results of the CESAR trial [5] and favorable outcome in A(H1N1) influenza-induced ARDS [6].

Since ECMO therapy requires highly specialized staff and equipment, an appropriate selection of patients stratified by outcome prediction for this economically costly therapy would be of great interest. This approach has been addressed by Pappalardo and colleagues who developed the ECMOnet score concerning risk stratification in H1N1 pneumonia [7] and Lindskov and colleagues who developed a score concerning ICU survival [8]. Recently, the question of 6-month outcome prediction and risk stratification of ARDS patients treated by ECMO was addressed by Schmidt and colleagues, who developed the Predicting Death for Severe ARDS on VV-ECMO (PRESERVE) score [9]. This mortality risk score was developed with eight pre-ECMO parameters; that is, age, body mass index, immunocompromised status, prone position, days of mechanical ventilation, sepsis-related organ failure assessment (SOFA), positive end-expiratory pressure (PEEP), and plateau pressure. Recently, the same group developed the Respiratory Extracorporeal membrane oxygenation Survival Prediction (RESP) score addressing the question of survival prediction after ECMO for severe acute respiratory failure [10]. Twelve pre-ECMO parameter were incorporated in this more complex score, where the acute respiratory diagnosis, central nervous dysfunction, acute associated non-pulmonary infection, neuromuscular blockade agents, use of NO, bicarbonate infusion, cardiac arrest, partial pressure of carbon dioxide (PaCO_2_) and peak inspiratory pressure are used in addition to age, immunocompromised status and days of mechanical ventilation.

Roch and colleagues [11] recently developed a simple score based on age, SOFA and diagnosis of influenza pneumonia for outcome prediction of ARDS patients selected for retrieval by a mobile ECMO team. The aim of the present study was to conduct an external validation of the PRESERVE score [9], the RESP score [10] and the score developed by Roch and colleagues [11] to investigate their usefulness in predicting survival in a single ECMO center.

## Material and methods

### Study design and objectives

All consecutive ARDS patients who received ECMO therapy in the ICUs of the University Hospital of Zurich between 1 January 2008 and 31 March 2013 were included.

### Patients

Criteria for ECMO were standardized according to local guidelines. ECMO therapy was evaluated for patients with severe but potentially reversible respiratory failure with persistent hypoxemia or hypercapnea. This was defined as a Horowitz index (PaO_2_/FiO_2_) <70 mmHg with FiO_2_ > 0.8 and/or pH <7.2 despite optimized mechanical ventilation (PEEP ≥10 cmH_2_O, tidal volume 6 ml/kg body weight) and possible use of adjunctive therapy such as NO, prone position, kinetic therapy and extracorporeal carbon dioxide removal systems. Case selection of patients with preceding mechanical ventilation of more than 7 days duration, high pressure (peak inspiratory pressure >30 cmH_2_O), FiO_2_ > 0.8 and patients over 65 years of age was performed based on the clinical opinion of the ECMO consultants.

Contraindications for ECMO therapy were age >75 years, pH <6.8, potassium >10 mmol/l, malignancies with fatal prognosis within 5 years, intracranial pathology or any other contraindication for therapeutic anticoagulation or patients with the decision for limitation of therapeutic interventions.

ECMO was performed primarily in a veno-venous (v-v) configuration. Veno-arterial (v-a) cannulation was chosen in selected patients when v-v ECMO was anticipated to be not sufficient enough, such as in cases of moderate to severe heart failure, severe hypoxemia, hemodynamic instability or pulmonary hypertension.

All v-v ECMO was performed with percutaneous cannulation. The standard configuration for v-v ECMO was femoro-jugular, and for v-a ECMO it was femoro-subclavian. An end-to-side anastomosis onto the right subclavian artery was made with an 8-mm vascular prosthesis and the cannula placed inside the graft. Circuit configuration was as follows: FemFlex cannula (Edwards Lifescience, Irvine, California, USA), heparin-coated tubing (Bioline, Maquet, Hirrlingen, Germany), ROTAFLOW centrifugal pumps (Maquet) and Quadrox PLS oxygenator with heater unit HU 35 (both Maquet).

### Data collection

We collected baseline demographic characteristics as well as physiologic and respiratory data immediately prior to initiation of ECMO therapy. Ventilation therapy and the use of precedent adjunctive therapy were noted. Data for hospital and ICU admission (local ICU and referral to ECMO center ICU) as well as the onset of mechanical ventilation were recorded. Laboratory data closest to the initiation of ECMO therapy were noted. Data for hospital survival and 6-month survival after ICU discharge were collected.

The PRESERVE score, RESP score and score according to Roch and colleagues were calculated according to the original papers [9-11] (Tables 1, 2 and 3). While the PRESERVE score was proposed for the risk estimation of death 6 months after ICU discharge for ARDS patients treated on ECMO, the RESP score and the score proposed by Roch and colleagues were developed for prediction of hospital mortality. Since plateau pressure (incorporated in the PRESERVE score) is not routinely determined in pressure-controlled ventilation in our institution, the PRESERVE scoring was adapted - peak inspiratory pressure expressing dynamic compliance was used as a surrogate for plateau pressure.

**Table 1:** **PRESERVE score parameters** [9]

**Parameter**		**Points**
Age (years)	<45	0
	45-55	2
	>55	3
Body mass index	>30 kg/m^2^	−2
Immunocompromised^a^		2
Mechanical ventilation	>6 days	1
SOFA	>12	1
No prone positioning before ECMO		1
PEEP	<10 cmH_2_O	1
Peak inspiratory pressure^b^	>35 cmH_2_O	1

^a^Defined as hematological malignancy, solid tumor, solid organ transplantation, high-dose or long-term corticosteroid and/or immunosuppressant use or HIV infection. ^b^Or plateau pressure >30 cmH_2_O = 1 point as proposed by Schmidt and colleagues [9]. ECMO, extracorporeal membrane oxygenation; PEEP, positive end-expiratory pressure; PRESERVE, Predicting Death for Severe ARDS on VV-ECMO; SOFA sepsis-related organ failure assessment.

**Table 2:** **RESP score parameters** [10]

**Parameter**	**Score**
Age (years)	
18-49	0
50-59	−2
≥60	−3
Immunocompromised status^a^	−2
Mechanical ventilation prior to initiation of ECMO	
<48 hours	3
48 hours to 7 days	1
>7 days	0
Acute respiratory diagnosis group (select only one)	
Viral pneumonia	3
Bacterial pneumonia	3
Asthma	11
Trauma and burn	3
Aspiration pneumonitis	5
Other acute respiratory diagnoses	1
Nonrespiratory and chronic respiratory diagnoses	0
Central nervous system dysfunction^b^	−7
Acute associated (nonpulmonary) infection^c^	−3
Neuromuscular blockade agents before ECMO	1
Nitric oxide use before ECMO	−1
Bicarbonate infusion before ECMO	−2
Cardiac arrest before ECMO	−2
PaCO_2_ (mmHg)	
<75	0
≥75	−1
Peak inspiratory pressure (cmH_2_O)	
<42	0
≥42	−1
Total score	−22 to 15

^a^Immunocompromised is defined as hematological malignancies, solid tumor, solid organ transplantation, human immunodeficiency virus, and/or cirrhosis. ^b^Central nervous system dysfunction diagnosis combined neurotrauma, stroke, encephalopathy, cerebral embolism, and seizure and epileptic syndrome. ^c^Acute associated (nonpulmonary) infection is defined as another bacterial, viral, parasitic, or fungal infection that did not involve the lung. An online calculator is available at [18]. ECMO, extracorporeal membrane oxygenation; PaCO_2_, partial pressure of carbon dioxide; RESP, Respiratory ECMO Survival Prediction.

**Table 3:** **Hospital mortality score proposed by Roch and colleagues** [11]

**Parameter**	**Points**
SOFA	
<9	0
9-11	1
≥12	2
Age (years)	
<45	0
≥45	1
Influenza pneumonia	
Yes	0
No	1
Total score	0-4

ECMO, extracorporeal membrane oxygenation; SOFA, sequential organ failure assessment.

The PRESERVE score [9] was originally defined for patients mainly treated on v-v ECMO (95%). Since v-a cannulation was applied in 29% of patients at our institution during the study period, an additional separate analysis of patients on v-v ECMO and v-a ECMO was chosen for better comparability of data. The same approach was chosen for the RESP score [10]. Only six patients in our study were initiated on ECMO in a referring hospital by a mobile ECMO team. Therefore, the score as proposed by Roch and colleagues [11] was applied for the whole study group.

This retrospective analysis was approved by the Kantonale Ethikkommission Zurich (KEK-ZH-Nr.2014-0318) which waived the need for informed consent for the study period.

### Statistical analysis

Data were tested for normal distribution using the Kolmogorov-Smirnov and Shapiro-Wilk tests. Continuous variables were analyzed using the Student’s *t*-test or Mann-Whitney *U* test as appropriate. Categorical variables were compared using Pearson’s Chi Square test or Fisher’s exact test as appropriate. All *P* values were two-sided and considered statistically significant if *P* ≤ 0.05. The cut-off value of 35 cmH_2_O for peak inspiratory pressure was determined by receiver operating curve (ROC) analysis and Yuden’s index (data not shown).

The discriminative performance of the calculated scores was evaluated by ROC analysis.

Statistical analysis was performed using SPSS Statistics version 22 (IBM, Chicago, IL, USA).

## Results

During the study period a total of 59 patients suffering from severe ARDS received ECMO therapy. Due to incomplete data sets, 51 patients were eligible for analysis; of these, 36 patients (71%) underwent v-v ECMO support and 15 patients (29%) underwent v-a ECMO support. V-v ECMO was performed as femoro-jugular cannulation in 72% of cases, while 28% were cannulated bifemorally. V-a ECMO was performed as femoro-subclavian cannulation in 60% of cases, while 40% were cannulated femoro-femoral.

Overall 6-month survival was 55% (95% CI 44 to 74%). Analyzing survival according to cannulation modus, v-v cannulated patients had a 6-month survival of 61% (95% CI 45 to 75%) while survival for v-a cannulated patients was 40% (95% CI 20 to 64%). This difference was not statistical significant (*P* = 0.22).

Patient baseline characteristics are presented in Table 4. Overall, the leading cause of ECMO implantation was bacterial infection followed by influenza and primary ARDS. Comorbidities as expressed by Charlson’s score [12] were more severe in the non-survivor group (*P* = 0.03), while age (*P* = 0.05) as well as the other tested variables did not reveal any statistically significant difference. Comparing baseline characteristics according to v-v or v-a cannulation modus did not reveal any difference between groups (see Additional file 1).

**Table 4:** **Baseline characteristics of all ECMO-treated ARDS patients according to survival status 6 months post-ICU discharge**

**Characteristic**	**All patients (n = 51)**	**Status 6 months post-ICU**		***P*value**
		**Alive (n = 28)**	**Dead (n = 23)**	
Age (years)	48 (33-58)	42 (31-53)	56 (38-60)	0.05
Men	27 (53)	16 (57)	11 (48)	0.35
Body mass index (kg/m^2^)	25 (22-29)	26 (23-29)	24 (20-32)	0.36
Charlson score	2 (1-4)	1 (1-4)	4 (2-5)	0.03
SAPS II	48 (30-61)	43 (29-56)	51 (39-67)	0.09
SOFA score	12 (9-13)	11 (9-13)	12 (9-15)	0.09
Chronic lung disease	8 (16)	4 (14)	4 (17)	0.76
Pregnant or postpartum	0 (0)	0 (0)	0 (0)	1.0
Diabetes mellitus	7 (14)	3 (11)	4 (17)	0.69
Renal insufficiency	4 (8)	3 (11)	1 (4)	0.62
Immunocompromised^a^	17 (33)	7 (25)	10 (44)	0.23
Hematological malignancies	4 (8)	3 (11)	1 (4)	0.32
Solid tumor	4 (8)	1 (4)	3 (13)	0.32
Solid organ transplantation	3 (6)	2 (7)	1 (4)	0.67
High-dose or long-term CS/IS	9 (18)	4 (14)	5 (22)	0.49
HIV	3 (6)	2 (7)	1 (4)	0.67
ARDS etiology				0.27
Peri-/postoperative	5 (10)	3 (11)	2 (9)	
Influenza A H_1_N_1_	8 (16)	2 (7)	6 (26)	
Influenza other	7 (14)	3 (11)	4 (17)	
Bacterial infection	21 (41)	13 (47)	8 (35)	
Primary ARDS	8 (16)	5 (18)	3 (13)	
Others	3 (6)	2 (7)	1 (4)	

Values are expressed as median (interquartile range) or n (%). ^a^Immuncompromised status included hematological malignancies, solid tumors, solid-organ transplantation, high-dose or long-term corticosteroids and/or immunosuppressant use, or HIV infection. ARDS, acute respiratory disease syndrome; CS/IS, corticosteroids or immunosuppressants; ECMO, extracorporeal membrane oxygenation; SAPS, simplified acute physiology score; SOFA, sepsis-related organ failure assessment.

Clinical and respiratory characteristics at ECMO initiation are presented in Table 5. Overall, time intervals between hospital admission and referral to the ECMO center (*P* = 0.046), hospital admission and ECMO implantation (*P* = 0.03), and ICU admission and ECMO implantation (*P* = 0.03) showed a significant difference between survivors and non-survivors. The same time intervals remained statistically significant in the sub-analysis of patients receiving v-v ECMO: non-survivors had a longer time interval between hospital admission and ECMO center referral (median 2 days (interquartile range (IQR) 1-4) versus median 8 days (IQR 7-12), *P* = 0.04), a longer time interval between hospital admission and ECMO implantation (median 4 days (IQR 2-8) versus 12 days (IQR 8-15), *P* < 0.001) and longer time interval between ICU admission and ECMO implantation (median 3 days (IQR 1-6) versus 8 days (IQR 7-11), *P* = 0.002). In the v-v group, the duration of mechanical ventilation before ECMO initiation was significantly longer in the non-survivor group (median 1 day (IQR 1-6) versus median 8 days (IQR 2-10), *P* = 0.02). In the v-a ECMO group, no difference between survivors and non-survivors could be detected. Comparing v-v and v-a cannulated patients, all collected parameters did not show any statistical significance between groups (see Additional file 2).

**Table 5:** **Clinical and ventilation characteristics at the time of ECMO initiation according to survival status**

**Characteristic**	**All patients (n = 51)**	**Status 6 months post-ICU**		***P*value**
		**Alive (n = 28)**	**Dead (n = 23)**	
Ventilation parameters				
PaO_2_/FiO_2_	57 (49-74)	55 (44-67)	64 (52-82)	0.049
FiO_2_	100 (100-100)	100 (100-100)	100 (100-100)	0.99
PEEP (cmH_2_O)	12 (10-15)	13 (10-15)	12 (10-15)	0.73
Tidal volume (ml/PBW kg)	8 (5-8)	7 (5-8)	8 (5-9)	0.51
Respiratory rate (per minute)	28 (24-33)	30 (24-36)	26 (23-31)	0.20
Peak inspiratory pressure (cmH_2_O)	34 (31-37)	33 (31-37)	34 (29-37)	0.70
Pre-ECMO blood gases				
pH	7.27 (7.12-7.35)	7.24 (7.11-7.31)	7.29 (7.15-7.37)	0.33
PaO_2_ (mmHg)	57 (49-73)	55 (44-67)	63 (52-74)	0.20
PaCO_2_ (mmHg)	57 (47-76)	57 (48-76)	58 (47-76)	0.85
HCO_3_^−^ (mmol/l)	24 (18-29)	24 (19-29)	23 (18-29)	0.88
SaO_2_ (%)	86.6 (78.0-92.7)	87 (76-93)	86 (82-92)	0.80
Lactate arterial (mmol/l)	1.6 (0.7-2.9)	1.6 (0.9-2.8)	1.6 (0.7-3.0)	0.75
Rescue therapy				
Prone positioning	9 (18)	3 (11)	6 (26)	0.27
Nitric oxide	26 (51)	15 (54)	11 (48)	0.78
Bilateral infiltration	48 (94)	27 (96)	21 (91)	0.58
Pre-ECMO steroids	23 (45)	11 (39)	12 (52)	0.41
Pre-ECMO vasopressors	46 (90)	25 (89)	21 (91)	0.81
Pre-ECMO pneumothorax	6 (12)	2 (7)	4 (17)	0.39
Mobile ECMO team	6 (12)	3 (11)	3 (13)	0.80
Interval (days)				
Hospital to ICU admission	1 (0-3)	1 (0-3)	1 (0-4)	0.89
Hospital to ECMO center admission	4 (1-8)	2 (1-6)	7 (4-10)	0.046
Hospital admission to ECMO	7 (4-13)	4 (2-13)	9 (6-14)	0.03
ICU admission to ECMO	4 (1-8)	3 (1-6)	7 (3-10)	0.03
MV to ECMO	2 (1-8)	1 (1-6)	4 (1-10)	0.14

Values are expressed as median (interquartile range) or n (%). ECMO, extracorporeal membrane oxygenation; FiO_2_, fraction of inspired oxygen; HCO_3_^–^, bicarbonate; MV, mechanical ventilation; PaO_2_, partial pressure of oxygen; PaCO_2_, partial pressure of carbon dioxide; PBW, predicted body weight; PEEP, positive end-expiratory pressure; SaO_2_, oxygen saturation.

The distribution of patients into groups according to the criteria of the PRESERVE and RESP groups, as well as by the score of Roch and colleagues, is presented in Tables 6, 7 and 8; ROC analysis of the scores is presented in Table 9. Data concerning the survival probabilities according to the various scores are presented in Table 10.

**Table 6:** **PRESERVE groups for all patients and according to the type of cannulation, either veno-venous or veno-arterial ECMO**

**PRESERVE group**	**Veno-venous and veno-arterial ECMO**				**Veno-venous ECMO**				**Veno-arterial ECMO**			
	**All patients (n = 51)**	**Status 6 months post-ICU**		***P*value**	**All patients (n = 36)**	**Status 6 months post-ICU**		***P*value**	**All patients (n = 15)**	**Status 6 months post-ICU**		***P*value**
		**Alive (n = 28)**	**Dead (n = 23)**			**Alive (n = 22)**	**Dead (n = 14)**			**Alive (n = 6)**	**Dead (n = 9)**	
				0.03				0.01				0.53
1	17 (33)	11 (21)	6 (12)		9 (25)	7 (32)	2 (14)		8 (53)	4 (67)	4 (44)	
2	13 (26)	10 (20)	3 (6)		11 (31)	9 (41)	2 (14)		2 (13)	1 (17)	1 (11)	
3	16 (31)	6 (12)	10 (20)		12 (33)	6 (27)	6 (43)		4 (27)	0 (0)	4 (44)	
4	5 (10)	1 (2)	4 (8)		4 (11)	0 (0)	4 (29)		1 (7)	1 (17)	0 (0)	

Data are expressed as n (%). PRESERVE score calculation as described in Table 1. ECMO, extracorporeal membrane oxygenation; PRESERVE, Predicting Death for Severe ARDS on VV-ECMO.

**Table 7:** **RESP Risk Groups for all patients and according to the type of cannulation, ether veno-venous or veno-arterial ECMO**

**RESP risk group**	**Veno-venous and veno-arterial ECMO**				**Veno-venous ECMO**				**Veno-arterial ECMO**			
	**All patients (n = 51)**	**Status at hospital discharge**		***P*value**	**All patients (n = 36)**	**Status at hospital discharge**		***P*value**	**All patients (n = 15)**	**Status at hospital discharge**		***P*value**
		**Alive (n = 28)**	**Dead (n = 23)**			**Alive (n = 22)**	**Dead (n = 14)**			**Alive (n = 6)**	**Dead (n = 9)**	
				0.07				0.035				0.61
I	3 (6)	3 (6)	0 (0)		3 (8)	3 (8)	0 (0)		0 (0)	0 (0)	0 (0)	
II	18 (35)	11 (22)	7 (14)		10 (28)	9 (25)	1 (3)		8 (53)	2 (13)	6 (39)	
III	23 (45)	13 (25)	10 (20)		17 (47)	9 (25)	8 (22)		6 (39)	4 (27)	2 (13)	
IV	7 (14)	2 (4)	5 (10)		6 (16)	2 (5)	4 (10)		1 (7)	0 (0)	1 (7)	
V	0 (0)	0 (0)	0 (0)		0 (0)	0 (0)	0 (0)		0 (0)	0 (0)	0 (0)	

Data are expressed as n (%). RESP score calculation as described in Table 2. ECMO, extracorporeal membrane oxygenation; RESP (score), Respiratory Extracorporeal membrane oxygenation Survival Prediction (score).

**Table 8:** **Roch Score for all patients and patients retrieved by mobile ECMO team only**

**Roch score**	**All patients (n = 51)**	**Status at hospital discharge**		***P*value**	**Externally retrieved patients (n = 6)**	**Status at hospital discharge**		***P*value**
		**Alive (n = 29)**	**Dead (n = 22)**			**Alive (n =3)**	**Dead (n =3)**	
				0.57				0.2
0-2	22 (43)	14 (27)	8 (16)		2 (33)	2 (33)	0 (0)	
3-4	29 (57)	15 (29)	14 (27)		4 (66)	1 (17)	3 (50)	

Data are expressed as n (%). Roch score calculation as described in Table 3. ECMO, extracorporeal membrane oxygenation.

**Table 9:** **ROC Analysis of the PRESERVE score, RESP score and the score published by Roch and colleagues**

**Score**		**Original publication**		**All patients (n = 51)**		**v-v ECMO (n = 36)**	
		**AUC**	**95% CI**	**AUC**	**95% CI**	**AUC**	**95% CI**
PRESERVE		0.89	0.83-0.94	0.67	0.52-0.82	0.75	0.57-0.92
RESP		0.74	0.72-0.76	0.65	0.5-0.8	0.81	0.67-0.95
	with PRESERVE population	0.92	0.89-0.97				
				**All patients (n = 51)**			
Roch		0.802	0.71-0.89	0.55	0.38-0.71		

AUC, area under the curve; PRESERVE, Predicting Death for Severe ARDS on VV-ECMO; RESP (score), Respiratory Extracorporeal membrane oxygenation Survival Prediction (score); ROC, receiver operating curve; v-v ECMO, veno-venous extracorporeal membrane oxygenation.

**Table 10:** **Survival according to the PRESERVE, RESP and Roch scores**

**Score**	**Group**	**Score**	**Original publication**	**All patients (n = 51)**	**v-v ECMO (n = 36)**	**v-a ECMO (n = 15)**
PRESERVE	1	0-2	97	65	78	50
	2	3-4	79	77	82	50
	3	5-6	54	38	50	0
	4	≥7	16	20	0	100
RESP	I	≥6	92	100	100	-
	II	3-5	76	61	90	25
	III	−1 to 2	57	56	53	30
	IV	−5 to −2	33	29	33	0
	V	≤–6	18	-	-	-
				**All patients (n = 51)**	**Mobile ECMO (n = 6)**	
Roch	-	0-2	60	64	100	-
	-	3-4	7	50	25	-

Results are shown as %. PRESERVE, Predicting Death for Severe ARDS on VV-ECMO; RESP (score), Respiratory Extracorporeal membrane oxygenation Survival Prediction (score); v-v ECMO, veno-venous extracorporeal membrane oxygenation; v-a ECMO, veno-arterial extracorporeal membrane oxygenation.

**Figure 1:**
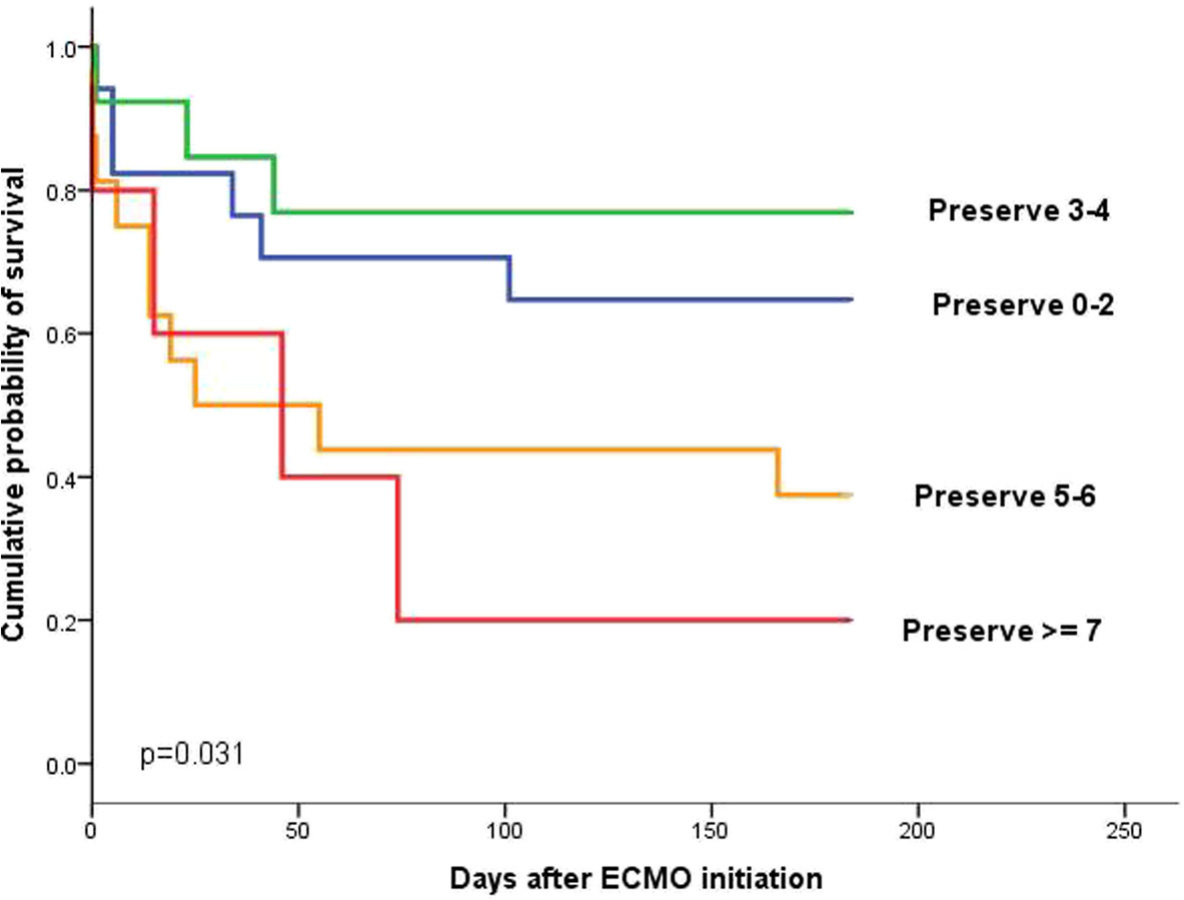
**Kaplan-Meier estimates of cumulative probabilities of 6-month survival for patients in PRESERVE groups 1 (0-2 points, n = 17), 2 (3-4 points, n = 13), 3 (5-6 points, n = 16) and 4 (≥7 points, n = 5).** ECMO, extracorporeal membrane oxygenation; PRESERVE, Predicting Death for Severe ARDS on VV-ECMO.

Overall, the PRESERVE score significantly discriminated survivors and non-survivors with an area under the curve (AUC) of 0.67 (95% CI 0.52 to 0.82; *P* = 0.03). Survival when subdivided into groups according to the PRESERVE score is presented in Figure 1. For patients on v-a ECMO the PRESERVE score failed to predict survival (*P* = 0.96). The PRESERVE score showed the best performance for patients on v-v ECMO (AUC = 0.75, 95% CI 0.57 to 0.92; *P* = 0.01). The RESP score performed similarly, with an AUC of 0.65 (95% CI 0.5 to 0.8) for all patients and the best performance for v-v cannulated patients (AUC = 0.81, 95% CI 0.67 to 0.95), but it was only statistically significant for the v-v group (*P* = 0.035).

Applying the scoring method developed by Roch and colleagues [11] for the whole study group did not discriminate between survivors and non-survivors (AUC = 0.55, 95% CI 0.38 to 0.71; *P* = 0.57).

## Discussion

This study is a retrospective analysis of 51 patients requiring ECMO support for severe ARDS in our institution, testing the recently proposed PRESERVE [9] and RESP [10] scores as well as the score developed by Roch and colleagues [11] for outcome prediction of ARDS patients treated on ECMO. The objective was to test the usefulness of these scoring systems for future identification of those ARDS patients likely to profit from ECMO therapy. The results indicate that both the PRESERVE score and the RESP score are of help identifying those patients suitable for ECMO therapy.

Comparing our baseline patients characteristics to Schmidt and colleagues [9,10] with respect to parameters incorporated in the PRESERVE score calculation, patients were comparable concerning age, body mass index, SOFA score, days of mechanical ventilation before ECMO and PEEP, while the incidence of immunocompromised patients and use of prone positioning before ECMO was lower in our study population. The lower incidence of prone positioning affects the score calculation by yielding higher scores and should be borne in mind when translating our findings into the clinic. We substituted the score by peak inspiratory pressure for plateau pressure score due to ventilation using the pressure-controlled mode (BiPAP®; Draeger, Lübeck, Germany) prior to ECMO implantation (peak pressure ≥35 cmH_2_O = 1 point).

Despite finding a similar PaO_2_/FiO_2_ ratio to Schmidt and colleagues [9], patients in our study were ventilated with a higher tidal volume and had a lower PaCO_2__._ This may be ascribed to the use of BiPAP® ventilation allowing spontaneous breathing in our study, while patients were ventilated in a volume-controlled mode in the study of Schmidt and colleagues [9].

With regards to parameters incorporated in the RESP score [10], patients were comparable concerning PaCO_2_, peak inspiratory pressure and days of mechanical ventilation before ECMO initiation, but patients in our study tended be older with a higher incidence of immunocompromised status. Data capture concerning bicarbonate infusion and neuromuscular blockade agents were incomplete in our study population (complete for 18% concerning bicarbonate and 49% concerning neuromuscular blockade agents), thereby influencing the score calculation.

Overall, the PRESERVE score is easier to use, while calculation of the RESP score (with a total of 12 different variables) is much more complex therefore limiting its bedside practicality.

Due to the very small number of patients that underwent initiation of ECMO in another hospital by our mobile ECMO team, we were not able to perform a validation of the score as proposed by Roch and colleagues for the original setting [11]. When applying the score to the whole study population, this score failed to predict hospital mortality.

A striking difference between our study and that of Schmidt and colleagues [9] is the number of patients treated on v-a ECMO. In our study, v-a cannulation was chosen in 29% of cases when deemed necessary because of severe hypoxemia, hemodynamic instability and pulmonary hypertension as assessed by echocardiography or pulmonary artery catheterization (irrespective of signs of moderate to severe cardiac failure). In contrast, Schmidt and colleagues [9] applied v-a ECMO in only 5% of patients with moderate to severe cardiac depression. A similarly high percentage of v-a cannulation in ARDS patients as in our study was reported by Hemmila and colleagues [13] who explained it as “need for systemic arterial perfusion support in addition to respiratory support”. Mortality rates in patients receiving v-a cannulation are higher in our study and in the study of Hemmila and colleagues [13] compared to v-v cannulated patients, suggesting that patients in whom v-a cannulation is considered represent a subset of patients with a worse prognosis. However, we cannot exclude that v-a cannulation by itself is a predictor for poor survival of unknown origin [14].

Overall 6-month survival of all patients included in our study was 55%. Survival for patients treated on v-v ECMO (61%) is comparable [5,9,15] or higher [12,16] than in previous studies. As in the studies of Schmidt and colleagues [9,10], survivors in our study were younger, had less comorbidity and a shorter time interval between admission and ECMO initiation.

Validating the PRESERVE and RESP scores in the setting of our study confirms their usefulness in discriminating survival probability for ARDS patients treated on ECMO in a tertiary hospital (Figure 1), and particularly in the subgroup of patients treated on v-v ECMO. Because of the small number of patients treated on v-a ECMO no final conclusion can be drawn from the findings in our study. In the subgroup of patients treated on v-v ECMO we found an AUC of 0.75 (95% CI 0.57 to 0.92) using the PRESERVE score and an AUC of 0.81 (95% CI 0.67 to 0.95) with the RESP score, compared to 0.89 (95% CI 0.83 to 0.94) in the PRESERVE study [9] and 0.74 (95% CI 0.72 to 0.76) in the RESP study [10]. The lower AUC of the PRESERVE score in our study suggests a weaker but significant performance for the PRESERVE score if applied externally, which is not the case for the RESP score. While there is good agreement of mortality risk estimation in PRESERVE score groups 2 and 3, the overall survival in group 1 in our study was lower than in the French study (78% versus 97%). In contrast, mortality risk estimation obtained using the RESP score is much closer to what we observed. This difference between PRESERVE mortality risk estimation and observed mortality may be explained by the high mortality in the subgroup of patients treated with v-a ECMO (60%) in our study, again suggesting that, compared to v-v cannulation, v-a cannulation is an unfavorable prognostic factor. The slightly higher Charlson’s comorbidity index in our patients may also contribute to the higher mortality. While the PRESERVE score is validated for use in ARDS patients through our findings, it showed suboptimal performance in a heterogeneous collective of patients treated on v-v ECMO for acute respiratory failure [17].

Recently, another publication [7] assessed the risk of unsuccessful ECMO treatment in severe ARDS patients. Based on the Italian national referral network set up by the health care authorities to face the H1N1 epidemic in 2009, the ECMOnet score has been created [7]. Length of stay in hospital and extra-pulmonary organ function before ECMO support were significant independent predictors of death.

Available study data [7,9,10], as well as the findings in our study, suggest that pulmonary infection (viral or bacterial), asthma, direct non-bacterial lung injury (trauma, burn, aspiration) and duration of mechanical ventilation <48 hours are predictors of a successful ECMO treatment in acute severe respiratory failure. In contrast, mechanical ventilation prior to ECMO treatment for more than 5 to 7 days, a non-pulmonary cause of ARDS, chronic lung disease, high PaCO_2_ (≥75 mmHg) and an elevated peak inspiratory pressure (>35 cmH_2_O) are independent predictors for poor ECMO treatment outcome characterizing the state of lung disease. Among non-pulmonary risk factors for poor outcome, older age (>60 years), immunocompromised status, central nervous system dysfunction, cardiac arrest before ECMO and SOFA score >12 were identified.

A limitation of our study is the retrospective design and the relatively small number of patients included, limiting the statistical power of the analysis. The high percentage of v-a cannulated patients in our study may have introduced an additional external confounder influencing our validation of the PRESERVE score. Due to the differences in cannulation modus and characteristics/outcomes in v-a and v-v cannulated ARDS patients, this topic should be addressed in further studies to identify factors guiding the choice of cannulation mode in these patients. Likewise, further characterization of patients with a low survival rate (PRESERVE group 4) would be valuable to guide decision finding before ECMO implantation.

## Conclusion

In conclusion, our study results validate the PRESERVE and RESP scores as a useful tool for risk stratification in patients suffering from severe ARDS considered for ECMO therapy. While the ECMOnet score [7] is more limited to the H1N1 experience, the PRESERVE score [9] and the RESP score [10] are valuable options for risk assessment in patients with severe respiratory failure. Since ECMO therapy is a highly invasive and costly therapy where optimal conditions for patient selection are required, this is an important finding for further improvement in stratification of mortality risk in these patients.

## Key messages

- The PRESERVE and RESP scores prove useful in this external validation.
- This is an important finding for further stratification of mortality risk for ECMO treatment in severe ARDS.

## Additional files

Additional file 1: **Baseline characteristics of extracorporeal membrane oxygenation (ECMO)-treated adult respiratory distress syndrome (ARDS) patients stratified by cannulation (veno-venous (v-v) or venous-arterial (v-a)) according to survival status at 6 months post-ICU.**

Additional file 2: **Clinical, ventilation and laboratory characteristics at the time of extracorporeal membrane oxygenation (ECMO) stratified by cannulation (veno-venous (v-v) or venous-arterial (v-a)) according to survival status at 6 months post-ICU.**

## Abbreviations

ARDS: adult respiratory distress syndrome
AUC: area under the curve
ECMO: extracorporeal membrane oxygenation
FiO_2_: fraction of inspired oxygen
IQR: interquartile range
NO: nitric oxide
PaCO_2_: partial pressure of carbon dioxide
PaO_2_: partial pressure of oxygen
PEEP: positive end-expiratory pressure
PRESERVE: Predicting Death for Severe ARDS on VV-ECMO
RESP (score): Respiratory Extracorporeal membrane oxygenation Survival Prediction (score)
ROC: receiver operating curve
SOFA: sepsis-related organ failure assessment
v-a: veno-arterial
v-v: veno-venous
