# Supplementary material for: External validation of scores proposed for estimation of survival probability of patients with severe adult respiratory distress syndrome undergoing extracorporeal membrane oxygenation therapy: a retrospective study
Source: Crit Care. 2015 Dec 1;19:142. doi: 10.1186/s13054-015-0875-z (PMC4403939; doi:10.1186/s13054-015-0875-z)
Supplement: Supplementary file 1 — Baseline characteristics of extracorporeal membrane oxygenation (ECMO)-treated adult respiratory distress syndrome (ARDS) patients stratified by cannulation (veno-venous (v-v) or venous-arterial (v-a)) according to survival status at 6 months post-ICU. [file 13054_2015_875_MOESM1_ESM.pdf]

Additional file 1. Baseline characteristics of ECMO-treated ARDS patients stratified by cannulation (v-v or v-a) according to survival status 6 months post-ICU

Characteristic	v-v ECMO (n=36)	v-a ECMO (n=15)	p value
Age (years)	48 (34-59)	48 (24-56)	0.35
Men	18 (50)	9 (60)	0.55
Body Mass Index (kg/m^2)	25 (22-29)	24 (19-31)	0.36
Charlson Score	3 (1-5)	2 (1-4)	0.51
SAPS II	45 (29-59)	58 (30-69)	0.24
SOFA score	12 (9-13)	11 (8-16)	0.96
Chronic lung disease	4 (11)	4 (27)	0.16
Pregnant or postpartum	0 (0)	0 (0)	1.0
Diabetes mellitus	5 (14)	2 (13)	0.95
Renal insufficiency	2 (6)	2 (13)	0.35
Immunocompromised[a]	14 (39)	3 (20)	0.19
Hematological malignancies	3 (8)	1 (7)	0.84
Solid tumor	3 (8)	1 (7)	0.84
Solid organ transplantation	2 (6)	1 (7)	0.88
High-dose or long-term CS/IS	7 (19)	2 (13)	0.60
Human immunodeficiency virus	3 (8)	0 (0)	0.25
ARDS etiology			0.08
Peri-/postoperative	5 (14)	0 (0)	
A H_1N_1 Influenza	4 (11)	4 (27)	
Influenza other	5 (14)	2 (13)	
Bacterial infection	8 (22)	0 (0)	
Others	14 (39)	9 (60)	

Values are expressed as median (interquartile range) or n (%).

[a]Immuncompromised status included hematological malignancies, solid tumors, solid-organ transplantation, high-dose or long-term corticosteroids and/or immunosuppressant use, or human immunodeficiency virus infection.

Abbreviations: ARDS Acute respiratory disease syndrome, CS/IS corticosteroids or immunosuppressants, ECMO extracorporeal membrane oxygenation, ICU intensive care unit, SAPS simplified acute physiology score, SOFA sepsis-related organ failure assessment
