# Supplementary material for: External validation of scores proposed for estimation of survival probability of patients with severe adult respiratory distress syndrome undergoing extracorporeal membrane oxygenation therapy: a retrospective study
Source: Crit Care. 2015 Dec 1;19:142. doi: 10.1186/s13054-015-0875-z (PMC4403939; doi:10.1186/s13054-015-0875-z)
Supplement: Supplementary file 2 — Clinical, ventilation and laboratory characteristics at the time of extracorporeal membrane oxygenation (ECMO) stratified by cannulation (veno-venous (v-v) or venous-arterial (v-a)) according to survival status at 6 months post-ICU. [file 13054_2015_875_MOESM2_ESM.pdf]

Additional file 2. Clinical, ventilation and laboratory characteristics at the time of ECMO stratified by cannulation (v-v or v-a) according to survival status 6 months post-ICU

Characteristic	v-v ECMO (n=36)	v-a ECMO (n=15)	p value
Ventilation parameters			
PaO_2/FiO_2	60 (51-76)	55 (42-68)	0.36
FiO_2	100 (100-100)	100 (100-100)	0.98
PEEP (cm H_2O)	12 (10-15)	12 (10-15)	0.51
Tidal Volume (mL/PBW kg)	8 (5-8)	8 (4-9)	0.96
Respiratory rate (min)	30 (24-34)	25 (20-31)	0.41
Peak inspiratory pressure (cm H_2O)	34 (31-37)	32 (30-38)	0.72
Pre-ECMO Blood gases			
pH	7.26 (7.18-7.34)	7.27 (7.03-7.39)	0.78
PaO_2 (mmHg)	60 (51-74)	55 (42-68)	0.38
$PaCO_2$ (mmHg)	57 (49-71)	57 (42-113)	0.69
HCO_3^- (mmol/l)	24 (19-29)	24 (17-29)	0.89
SaO_2 (%)	89 (80-93)	86 (62-91)	0.25
Lactate arterial (mmol/l)	1.5 (0.8-2.3)	1.9 (0.6-4.6)	0.55
Rescue therapy			
Prone positioning	7 (19)	2 (13)	0.60
Nitric oxide	18 (50)	8 (53)	0.83
Bilateral infiltration	35 (97)	13 (87)	0.14
Pre-ECMO Steroids	14 (39)	9 (60)	0.17
Pre-ECMO Vasopressors	33 (92)	13 (87)	0.62
Pre-ECMO Pneumothorax	3 (8)	3 (20)	0.24
Mobile ECMO-team	4 (11)	2 (13)	0.82
Interval (days)			
Hospital - ICU admission	1 (0-4)	1 (0-3)	0.41
Hospital-ECMO center admission	4 (2-10)	5 (1-7)	0.47
Hospital admission-ECMO	8 (4-13)	6 (4-18)	0.69

ICU admission - ECMO	4 (1-10)	3 81-6)	0.52
MV-ECMO	3 (1-8)	2 (1-6)	0.76

Values are expressed as median (interquartile range) or n (%).
Abbreviations: ECMO extracorporeal membrane oxygenation; ICU intensive care unit; MV mechanical ventilation; PBW predicted body weight.
